# Downregulation of USP18 reduces tumor-infiltrating activated dendritic cells in extranodal diffuse large B cell lymphoma patients

**DOI:** 10.18632/aging.203030

**Published:** 2021-05-17

**Authors:** Chong Zhao, Runzhi Huang, Zhiwei Zeng, Shaoxin Yang, Wei Lu, Jiali Liu, Yanyu Wei, Hezhou Guo, Yanjie Zhang, Penghui Yan, Zongqiang Huang, Jun Shi

**Affiliations:** 1 Department of Hematology, Shanghai Ninth People’s Hospital, Shanghai Jiao Tong University School of Medicine, Shanghai, China; 2 Division of Spine, Department of Orthopedics, Tongji Hospital Affiliated to Tongji University School of Medicine, Shanghai, China; 3 Key Laboratory of Spine and Spinal Cord Injury Repair and Regeneration (Tongji University), Ministry of Education, Shanghai, China; 4 Department of Orthopedics, The First Affiliated Hospital of Zhengzhou University, Zhengzhou, China; 5 Department of Hematology, Shanghai Jiao Tong University Affiliated Sixth People's Hospital, Shanghai, China

**Keywords:** extranodal diffuse large B cell lymphoma, USP18, MAPK pathway, dendritic cells

## Abstract

Extranodal diffuse large B cell lymphoma (EN DLBCL) often leads to poor outcomes, while the underlying mechanism remains unclear. As immune imbalance plays an important role in lymphoma pathogenesis, we hypothesized that immune genes might be involved in the development of EN DLBCL. Ninety-three differentially expressed immune genes (DEIGs) were identified from 1168 differentially expressed genes (DEGs) between tumor tissues of lymph node DLBCL (LN DLBCL) and EN DLBCL patients in TCGA database. Nine prognostic immune genes were further identified from DEIGs by univariate Cox regression analysis. A multivariate predictive model was established based on these prognostic immune genes. Patients were divided into high- and low-risk groups according to the median model-based risk score. Kaplan-Meier survival curves showed that patients in the high-risk group had a shorter survival time than those in the low-risk group (P < 0.001). Ubiquitin-specific peptidase 18 (USP18) was further recognized as the key immune gene in EN DLBCL on the basis of coexpression of differentially expressed transcription factors (DETFs) and prognostic immune genes. USP18 exhibited low expression in EN DLBCL, which was regulated by LIM homeobox 2 (LHX2) (R = 0.497, P < 0.001, positive). The potential pathway downstream of USP18 was the MAPK pathway, identified by gene set variation analysis (GSVA), gene set enrichment analysis (GSEA) and Pearson correlation analysis (R = 0.294, P < 0.05, positive). The “ssGSEA” algorithm and Pearson correlation analysis identified that activated dendritic cells (aDCs) were the cell type mostly associated with USP18 (R = 0.694, P < 0.001, positive), indicating that USP18 participated in DC-modulating immune responses. The correlations among key biomarkers were supported by multiomics database validation. Indeed, the USP18 protein was confirmed to be expressed at lower levels in tumor tissues in patients with EN DLBCL than in those with LN DLBCL by immunohistochemistry. In short, our study illustrated that the downregulation of USP18 was associated with reduced aDC number in the tumor tissues of EN DLBCL patients, indicating that targeting USP18 might serve as a promising therapy.

## INTRODUCTION

As the predominant subtype of non-Hodgkin lymphoma (NHL) worldwide, DLBCL accounts for 30-40% of lymphoid malignancies [1, 2]. DLBCLs often originate from lymph nodes, while up to one-third of DLBCLs occur in extranodal sites [3]. Specific primary sites, such as the CNS and breast, are often associated with worse outcomes [4–6], indicating that these two groups of DLBCL have separate clinical and biological characteristics. However, the distinction between the development of EN and LN DLBCL has not yet been fully clarified.

As important participants in immune responses, immune cells behave differently in the development of EN DLBCL and LN DLBCL. For example, the numbers of certain immune cells, such as regulatory T cells and macrophages, were significantly lower in primary CNS DLBCL than in systemic DLBCL [7]. Extranodal lymphomas also showed fewer tumor-associated CD45RO^+^ T cells and less conspicuous dendritic cell infiltration [8]. Abnormal function of immune genes might induce an imbalance in immune cells. Immune genes in cancer cells might promote the secretion of inflammatory factors such as chemokines by activating downstream pathways, recruiting immunosuppressive cells to repress immune killing and thus accelerating cancer progression [9]. DLBCL is a type of lymphoid malignancy that is caused by developmental blockage and uncontrolled proliferation of large lymphoid cells expressing B cell markers. Thus, the dysfunction of immune genes in B lymphoblasts might also lead to immune imbalance. However, how immune gene dysfunction contributes to immune imbalance in EN DLBCL remains elusive.

An immune gene set is a collection of immune genes associated with an immune response event. Currently, the ssGSEA tool is applied to identify immune gene sets in gene expression profiles from tumor tissues [10], aiming to explore immune response events or immune cells involved in tumor development. In this study, we identified the key immune genes in EN DLBCL from differentially expressed immune genes (DEIGs) between EN and LN DLBCL and then explored the downstream KEGG pathways and immune gene sets with gene set variation analysis (GSVA), gene set enrichment analysis (GSEA) and ssGSEA tools. Finally, the differential expression of the key immune genes was confirmed in the tumor tissues of LN and EN DLBCL patients by immunohistochemistry (IHC).

## RESULTS

### Nine prognostic immune genes were identified in EN DLBCL

**Figure 1:**
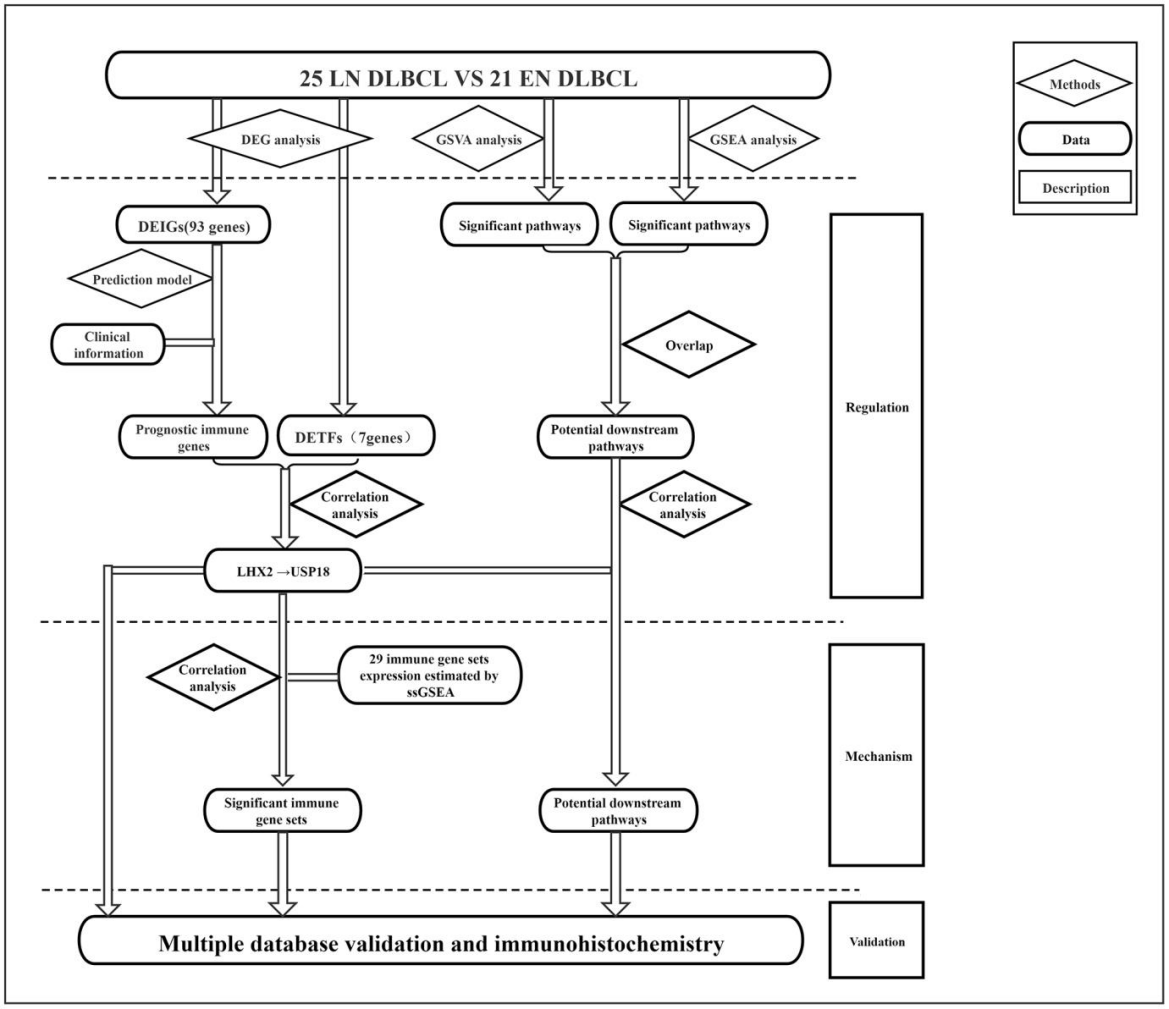
The flowchart of analysis process of this study.

**Figure 2:**
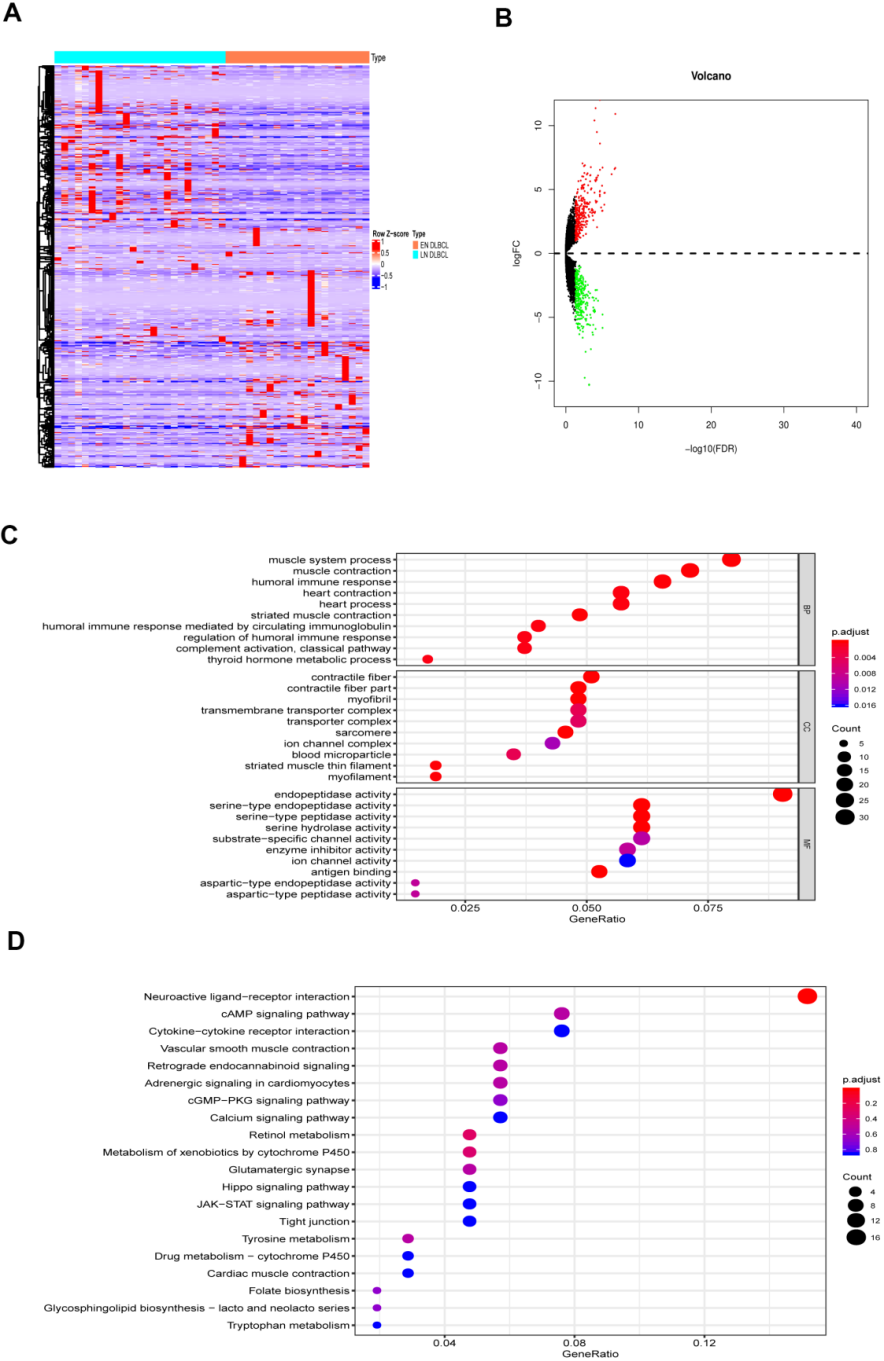
**The DEGs between LN DLBCL and EN DLBCL.** (**A**) The heatmap and (**B**) volcano plot of 1168 DEGs between 21 LN DLBCL and 25 EN DLBCL; (**C**) The GO and (**D**) KEGG analyses of 1168 DEGs. Abbreviations: DEGs, Differentially expressed genes; DLBCL, Diffuse large B-cell lymphoma; GO, Go Ontology; KEGG, Kyoto Encyclopedia of Genes and Genomes.

The analysis process is shown in Figure 1. To determine the key biomarker related to EN DLBCL, we analyzed RNA-seq profiles and clinical data from 46 DLBCL patients consisting of 25 LN DLBCL and 21 EN DLBCL from TCGA database. The baseline information of the samples is presented in Table 1. As shown in Figure 2A, 2B, DEGs consisting of 583 up- and 585 down-regulated genes between these two groups were illustrated by heatmap and volcano plot. Then, GO and KEGG enrichment analyses were performed to uncover the potential mechanism distinguishing between the development of LN and EN DLBCL. As shown in Figure 2C, 2D, immune-related pathways such as “cytokine-cytokine receptor interaction” and “JAK-STAT signaling pathway” were included in the top ten terms, indicating that immune-related mechanisms were involved in the developmental difference between LN and EN DLBCL.

**Table 1:** Baseline information of 46 patients with DLBCL from the TCGA database.

**Variables**	**Total patients (N=46)**
Age, years	
Mean ± SD	55.98 ± 26.02
Gender	
Female	25 (54.3%)
Male	21 (45.6%)
Race	
Asian	18 (39.1%)
Black or African American	1 (2.1%)
White	27 (58.6%)
Stage	
I-II	25 (54.2%)
III-IV	17 (36.8%)
Unknown	4 (8.6%)
Original location	
Extranodes	21 (45.6%)
Lymphnodes	25 (54.3%)
Outcome	
CR	34 (69.7%)
PR	2 (4.0%)
PD	4 (8.1%)
SD	2 (4.0%)
Unknown	7 (14.2%)

Abbreviations: SD, Standard deviation; CR, Complete Remission; PR, Partial Remission; PD, Progressive Disease; SD, Stable Disease; DLBCL, Diffuse large B-cell lymphoma.

**Figure 3:**
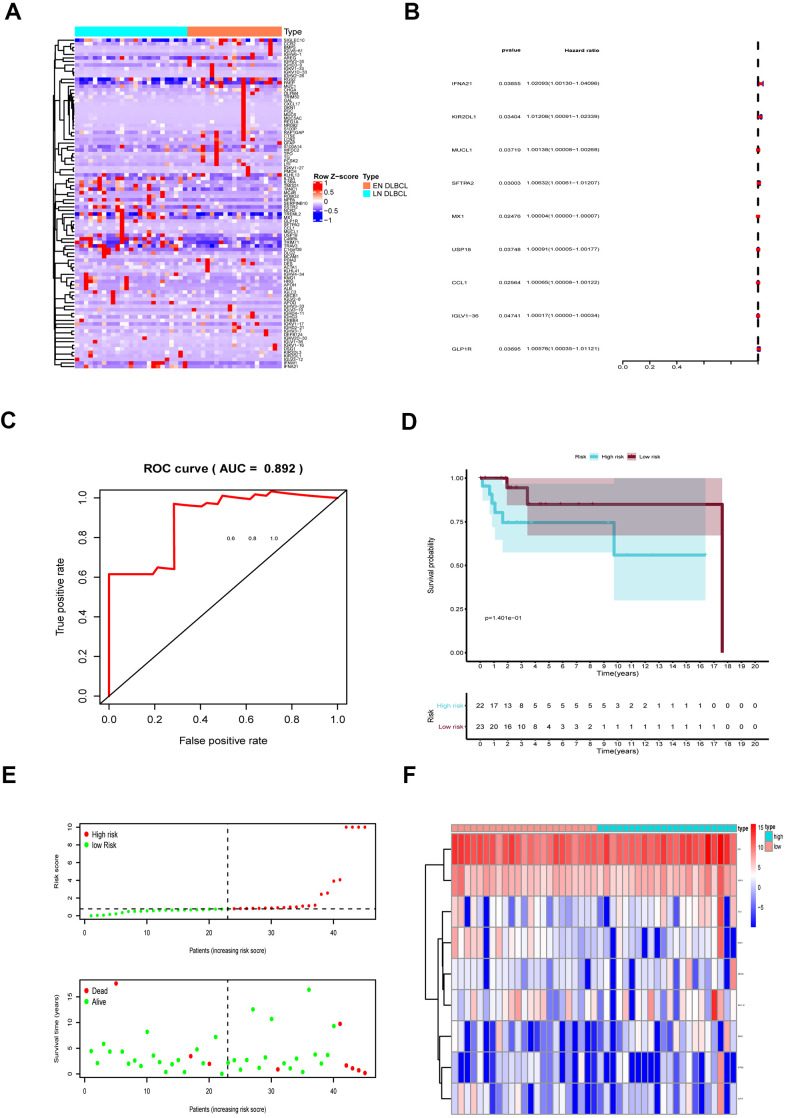
**The prognostic assessment model based on prognostic immune genes.** (**A**) The heatmap of 93 DEIGs; (**B**) Forest plot to show nine prognostic immune genes; Red: high-risk genes; Blue: low-risk genes; (**C**) The ROC to assess the prognostic model (AUC = 0.892); (**D**) The Kaplan-Meier curve to identify the efficacy of risk score; (**E**) The high- and low-risk score group in scatterplot and risk plot; (**F**) The heatmap to illustrate each prognostic immune gene screened by Lasso regression. Abbreviations: DEIGs, Differentially expressed immune genes; ROC, Receiver operator characteristic curve; AUC, Area under the curve.

The DEIGs were obtained by intersecting DEGs and immune-related genes. Ninety-three DEIGs, including 53 up- and 40 down-regulated DEIGs, are displayed in the heatmap in Figure 3A. Then, the DEIGs and prognostic characteristics were submitted to univariate Cox regression analysis to identify prognostic immune genes. As shown in Figure 3B, nine prognostic immune genes, including IFNA21, KIR2DL1, MUCL1, SFTPA2, MX1, USP18, CCL1, IGLV1-36, and GLP1R, were identified.

These nine genes were then integrated into the multivariate regression analysis to build a prognostic predictive model. To avoid overfitting of the predictive model, Lasso regression was performed. The AUC of the ROC curve was 0.892, indicating that all nine genes were essential for the model (Figure 3C). To further assess model fit, we also performed a Schoenfeld residuals test. As shown in Supplementary Figure 1A–1C, the slope of scaled residuals on time was zero, so the proportional hazards assumption in the Cox model conformed to the null hypothesis, indicating high accuracy of the model. According to the model, the risk score of each sample was calculated, and samples were divided into high- and low-risk groups with a median value of 0.786. As shown in Figure 3D, the Kaplan-Meier survival curve showed that patients in the high-risk group had a lower survival rate than those in the low-risk group (P < 0.001), further revealing the good effectiveness of the predictive model.

Then, we generated a risk curve and scatterplot to show the risk score and survival status of each individual with DLBCL. As shown in Figure 3E, patients in the high-risk group showed higher mortality than those in the low-risk group, which also indicated the high efficacy of the model. The expression of prognostic immune genes screened by Lasso regression is displayed by a heatmap in Figure 3F.

### USP18 was the key immune gene in EN DLBCL

**Figure 4:**
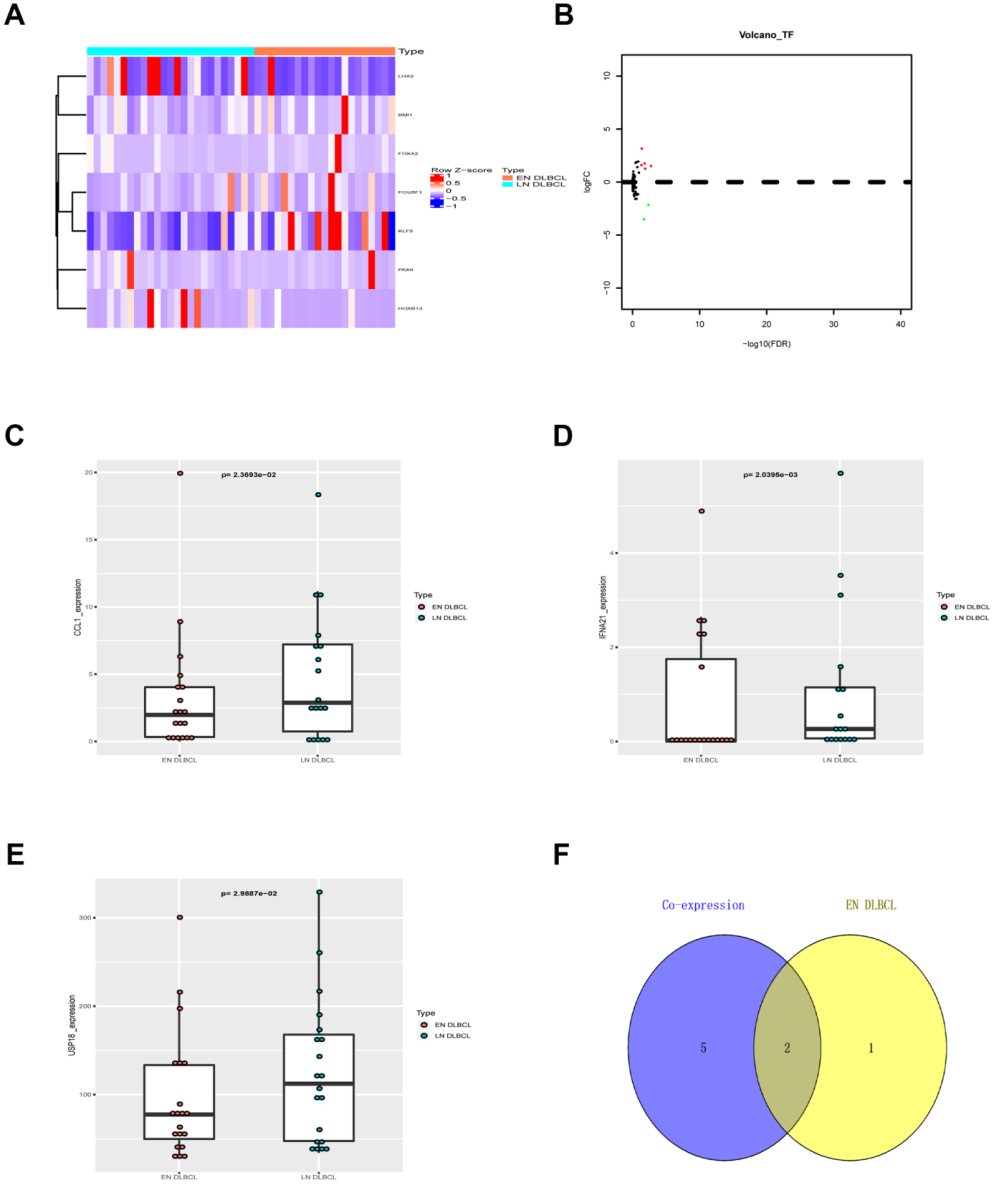
**The DETFs between EN and LN DLBCL.** (**A**) The heatmap and (**B**) volcano plot of 7 DETFs; (**C**) The box plot to show expression of CCL1 between EN and LN DLBCL; (**D**) The box plot to show expression of IFNA21 between EN and LN DLBCL; (**E**) The box plot to show expression of USP18 between EN and LN DLBCL; (**F**) The Venn plot to show overlap of ENI- and DETFs- related immune genes. Abbreviations: DETFs, Differentially expressed transcription factors; CCL1, C-C motif chemokine ligand 1; IFNA21, Interferon alpha-21; USP18, Ubiquitin specific peptidase 18; ENI, Extranodal involvement.

To further identify the key immune genes, the coexpression of DETFs and nine prognostic immune genes was performed. Five up- and two down-regulated DETFs were identified by intersecting cancer-associated TFs and DEGs, as shown in the heatmap and volcano plot (Figure 4A, 4B). The correlation analysis identified 7 regulatory pairs between DETFs and prognostic immune genes, as shown in Table 2. As shown in Figure 4C–4E, only the expression of CCL1, IFNA21 and USP18 from nine prognostic immune genes was significantly different between EN and LN DLBCL by the Wilcoxon test (P < 0.05). To identify the key immune genes related to EN DLBCL, DETF-related and extranodal involvement (ENI)-related immune genes were intersected. As shown in Figure 4F, two immune genes were found in both groups. Combined with the results of the correlation between DETFs and prognostic immune genes shown in Table 2, the regulatory pair of LIM homeobox 2 (LHX2) and ubiquitin-specific peptidase 18 (USP18) was most significant (R = 0.497, P < 0.001, positive). Consequently, USP18, which is downregulated in EN DLBCL compared with LN DLBCL, was recognized as the key immune gene.

**Table 2:** The correlation relationship between DETFs and prognostic immune genes.

**TF**	**Immune gene**	**Correlation**	**P-value**	**Regulation**
LHX2	KIR2DL1	0.447811649	0.002038224	positive
LHX2	MUCL1	0.439856042	0.002499669	positive
LHX2	SFTPA2	0.428968478	0.003279537	positive
LHX2	MX1	0.318302253	0.033095802	positive
LHX2	USP18	0.496825418	0.000517446	positive
LHX2	CCL1	0.452575151	0.001799553	positive
LHX2	GLP1R	0.531881138	0.000169843	positive

Abbreviations: DETFs, Differentially expressed transcription factors; KIR2DL1, Killer cell immunoglobulin like receptor, two Ig domains and long cytoplasmic tail 1; MUCL1, Mucin like 1; SFTPA2, Surfactant protein A2; MX1, MX dynamin like GTPase 1; USP18, Ubiquitin specific peptidase 18; CCL1, C-C motif chemokine ligand 1; GLP1R, Glucagon like peptide 1 receptor.

To demonstrate the regulatory mechanism between LHX2 and USP18, chromatin immunoprecipitation followed by high-throughput DNA sequencing (ChIP-Seq) data from the Cistrome database was evaluated. As shown in Supplementary Figure 2, *in vitro* ChIP-Seq data confirmed the transcriptional regulation patterns between LHX2 and USP18 in multiple cell lines.

### USP18 was positively associated with the MAPK pathway in EN DLBCL

**Figure 5:**
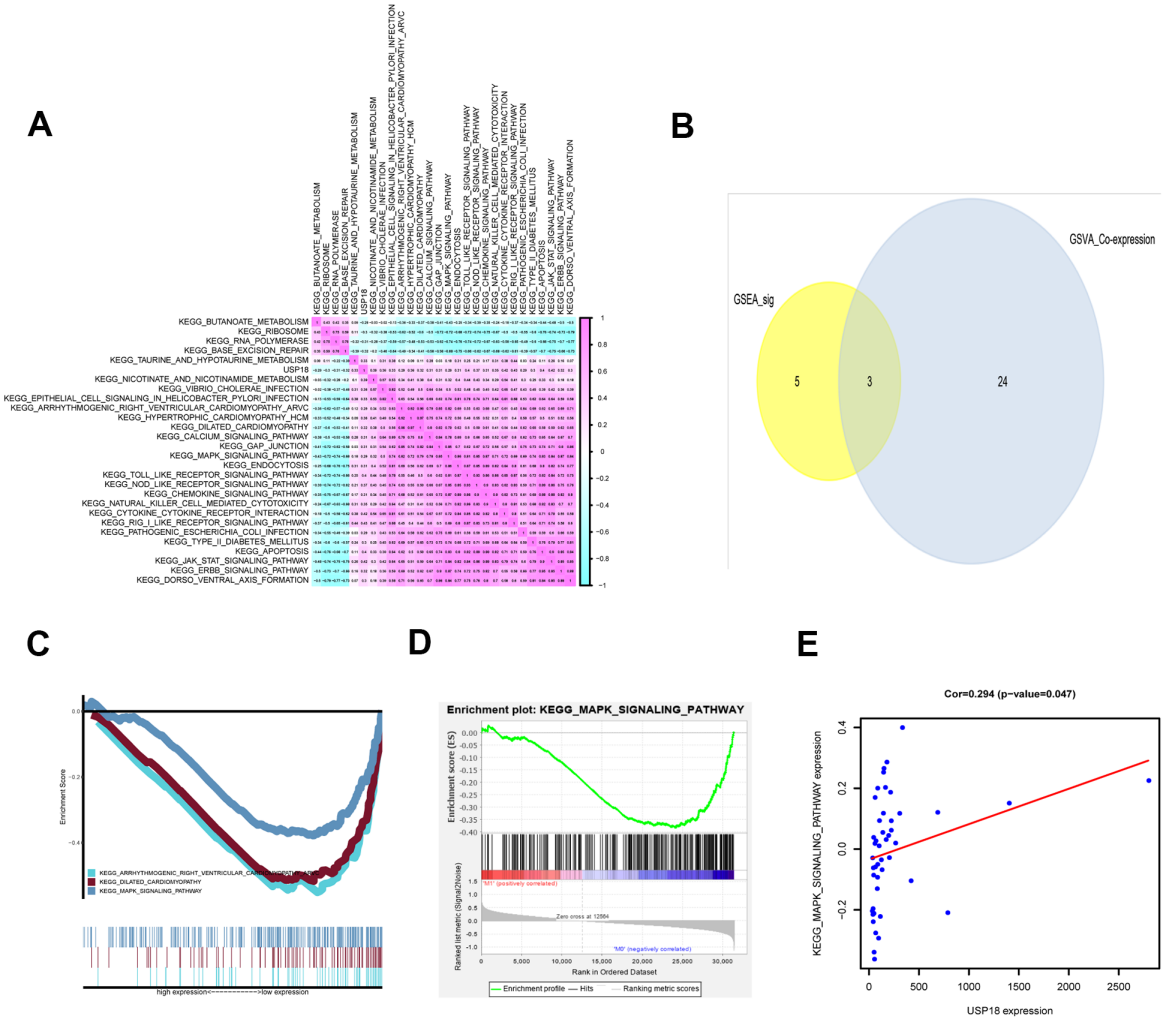
**The KEGG pathways downstream of USP18 in EN DLBCL.** (**A**) The coexpression heatmap of USP18 and KEGG pathways selected by GSVA. (**B**) The Venn plot to show overlapped KEGG pathways in both GSVA and GSEA; (**C**) The GSEA of overlapped KEGG pathways; (**D**) The GSEA of MAPK pathway; (**E**) The correlation between USP18 and MAPK signaling pathway. Abbreviations: GSVA, Gene set variation analysis; GSEA, Gene set enrichment analysis; MAPK, Mitogen-activated protein kinase.

To discover the pathway downstream of USP18, GSVA was conducted, and 27 KEGG signaling pathways between EN and LN DLBCL were identified. The correlations between USP18 and these 27 KEGG pathways were constructed by Pearson correlation analysis, as shown in Figure 5A. To determine the critical signaling pathway, GSEA was also conducted. Three key KEGG pathways, i.e., the arrhythmogenic right ventricular cardiomyopathy (ARVC) pathway, dilated cardiomyopathy pathway, and MAPK pathway, overlapped between GSEA and GSVA (Figure 5B, 5C). Considering the relevance to the disease, we focused on the MAPK pathway in the following analysis. The GSEA of the MAPK pathway is shown in Figure 5D. The correlation between USP18 and the MAPK pathway was fitted by linear regression. As shown in Figure 5E, USP18 was positively correlated with the MAPK pathway (R = 0.294, P < 0.05, positive), suggesting that USP18 might modulate the MAPK pathway in the development of EN DLBCL.

### Downregulation of USP18 in EN DLBCL was correlated with the immune gene set of aDCs

**Figure 6:**
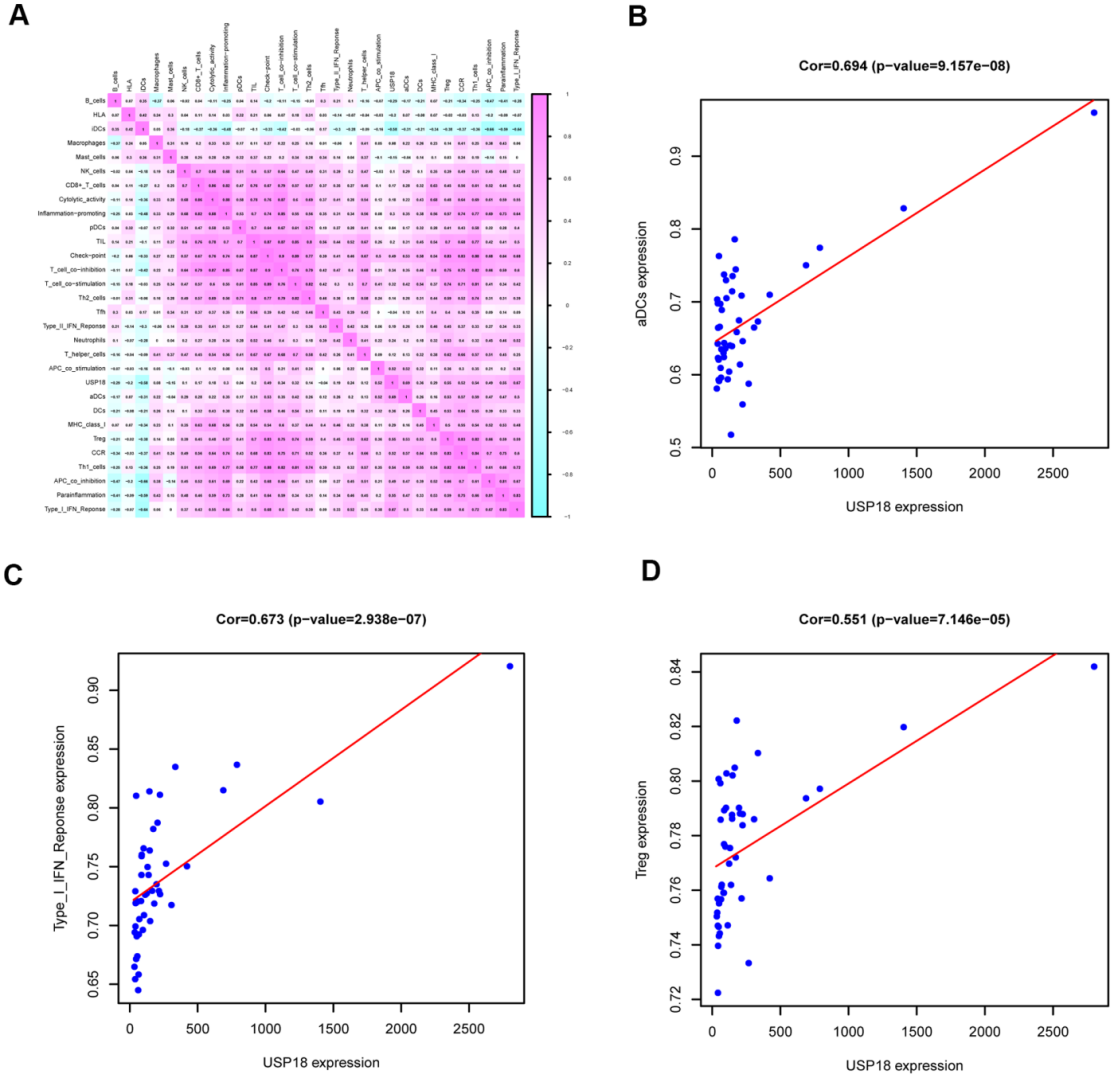
**The immune gene sets related to USP18 in EN DLBCL.** (**A**) The coexpression heatmap of USP18 with immune gene sets in DLBCL. (**B**) The linear regression to show the correlation between USP18 and aDCs; (**C**) The linear regression to show the correlation between USP18 and type I IFN response; (**D**) The linear regression to show the correlation between USP18 and Tregs; Abbreviations: aDCs, Activated dendritic cells; Tregs, Regulatory T cells.

To determine the immune responses involved in USP18, ssGSEA was applied. Fifteen immune gene sets were identified in DLBCL patients from 29 immune gene sets that were overexpressed in the tumor microenvironment [11]. Pearson correlation analysis between USP18 and immune gene sets in DLBCL was constructed, as shown by the heatmap in Figure 6A. The top three immune gene sets correlated with USP18 were aDCs (R = 0.694, P < 0.001, positive), type I IFN response (R = 0.673, P < 0.001, positive) and regulatory T cells (Tregs) (R = 0.551, P < 0.001, positive), as shown in Figure 6B–6D. Of these, the relationship between USP18 and aDCs was most significant, indicating that USP18 might affect aDCs in EN DLBCL.

### The online database further validated the association between key biomarkers in our analysis

To minimize the bias of the results above, a multidimensional validation was performed. The expression of LHX2 and USP18 and key genes of potential pathways in primary DLBCL, normal nodal tissue, and various cell lines, and their association with prognosis, are summarized in Supplementary Table 1.

First, LHX2 (median rank 695, P < 0.001), IL2RA (median rank 669, P < 0.001), IL21R (median rank 868, P < 0.001) and CHST7 (median rank 782, P < 0.001) were highly expressed in primary DLBCL compared to normal tissue, while IL5RA (median rank 3,659, P = 0.128) showed no difference in any of the four comparisons (Supplementary Figure 3). The GEPIA results showed that the mRNA expression levels of USP18, IL2RA and IL21R were higher in tumor samples than in normal samples (Supplementary Figure 6). At the cellular level, USP18, TLR7, IL21R, GCNT1 and CHST7 were expressed in various tumor cell lines, while the expression of LHX2, IL2RA and IL5RA was low in CCLE (Supplementary Figure 4). The results from The Protein Atlas showed the protein expression of USP18, IL2RA, TLR7, IL21R and GCNT1 in normal lymph node tissue (Supplementary Figure 10).

In addition, an analysis of the genomic and clinical profiles with cBioPortal suggested that LHX2, USP18 and key genes in downstream pathways were prone to mutations, which were associated with poor prognosis (Supplementary Figure 5A–5D). The results also showed that USP18 was coexpressed with LHX2 (R = 0.61, P < 0.001), IL5RA (R = 0.45, P < 0.001), IL21R (R = 0.56, P < 0.001), GCNT1 (R = 0.37, P = 0.024) and CHST7 (R = 0.43, P < 0.001) (Supplementary Figure 5E–5I). Moreover, analysis in the other databases also presented a negative association of key genes and prognosis (Supplementary Figures 7–9). Supplementary Figure 11 shows the PPI network of LHX2, USP18, IL2RA, IL5RA, IL21R, TLR7, GCNT1 and CHST7 generated in String.

### USP18 expression and the number of aDCs were low in EN DLBCL tumor tissues

**Figure 7:**
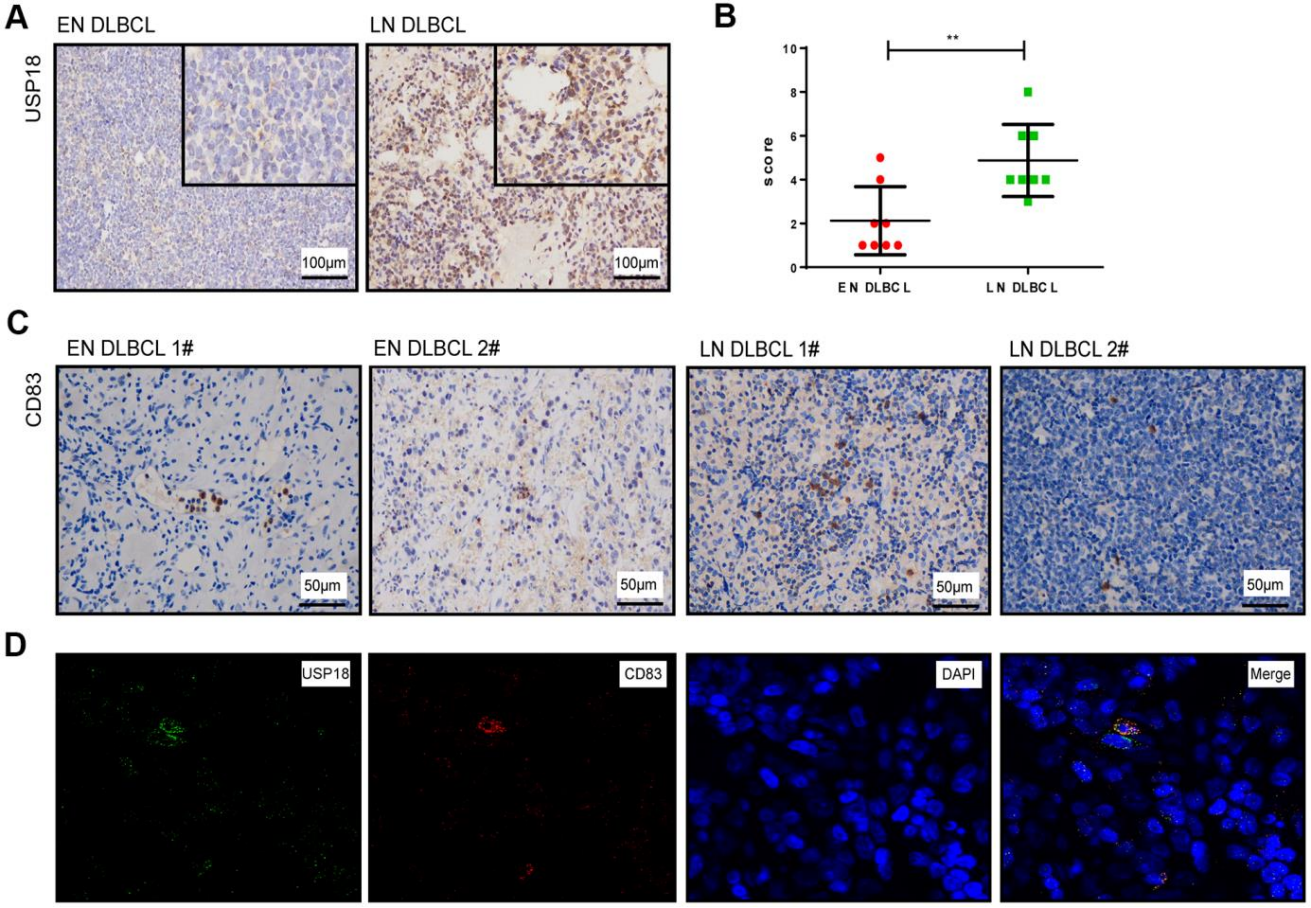
**The expression of USP18 protein in EN and LN DLBCL patients.** (**A**) The expression of USP18 protein in EN and LN DLBCL by IHC staining. (**B**) The H-score of USP18 in tumor tissues of EN and LN DLBCL. (**C**) The expression of CD83 protein in EN and LN DLBCL by IHC staining. (**D**) The immunofluorescence double labeled staining of USP18 and CD83 in DLBCL tissues.

To further verify the role of USP18, the expression of USP18 in tumor biopsies of patients with LN and EN DLBCL was detected by IHC staining. The clinical information of DLBCL patients is shown in Table 3. As shown in Figure 7A, the USP18 protein was expressed at lower levels in EN DLBCL tissues than in LN DLBCL tissues. Compared to that in patients with LN DLBCL, the H-score of USP18 in the tumor tissues of EN DLBCL patients was significantly lower (2.125 vs 5.625, P < 0.01) (Figure 7B). The IHC results further confirmed that the downregulation of USP18 expression was associated with EN DLBCL.

**Table 3:** Baseline information of 16 patients with DLBCL.

**Variables**	**LN DLBCL (N=8)**	**EN DLBCL (N=8)**
Age, years		
Mean ± SD	60±32	57.8±13.8
Gender		
Female	1	7
Male	7	1
Stage		
I-II	2	4
III-IV	6	4
IPI score		
Low risk	3	2
Intermediate/high risk	5	6

To show the distribution of aDCs in DLBCL tissues, we also detected the expression of the aDC marker CD83 on tumor biopsies of DLBCL patients. As shown in Figure 7C, the expression of CD83 was distributed throughout the tissues and was lower in EN DLBDL tissues than in LN DLBCL tissues.

Furthermore, to identify whether aDCs express USP18, we performed immunofluorescence double staining of USP18 with CD83 in DLBCL tissues. As shown in Figure 7D, USP18 was coexpressed with CD83, which indicated that DCs also expressed USP18.

## DISCUSSION

EN DLBCL often leads to poorer prognosis than LN DLBCL. Immunophenotypic, genetic and survival characteristics are related to the specific primary sites of the disease [12]. However, the mechanism underlying the development of EN DLBCL remains elusive. In the current study, we concluded that downregulation of the immune gene USP18 led to reduced aDC number, contributing to the development of EN DLBCL (Supplementary Figure 12).

In this study, we identified that LHX2 regulated the expression of USP18 in EN DLBCL by coexpression of DETFs and prognostic immune genes. With this approach, both key immune genes and their TFs were identified from the DEGs between EN and LN DLBCL, which might be a helpful step toward finding critical biomarkers. LHX2 is reported to participate in oncogenesis and promote tumor growth in breast cancer and pancreatic ductal adenocarcinoma [13, 14], suggesting the role of LHX2 in carcinogenesis and cancer progression. LHX2 is widely known for its transcriptional role in multiple biological processes [15–17]. However, no study has reported a direct regulatory relationship between LHX2 and USP18. In our study, ChIP-Seq data from the Cistrome database were analyzed, and the transcription regulation patterns between LHX2 and USP18 were confirmed. Furthermore, multidimensional validation in multiple online databases also confirmed the positive correlation relationship between LHX2 and USP18. Therefore, our results indicated that downregulation of LHX2 led to decreased expression of USP18 in EN DLBCL, although the details of their regulatory relationship need further experimental verification.

Our study identified USP18 as the key immune gene among nine prognostic immune genes in EN DLBCL. Moreover, the USP18 protein was confirmed to be expressed at low levels in tumor tissues of EN DLBCL patients by IHC staining. The USP18 protein belongs to a large family of ubiquitin-specific proteases (UBPs). It cleaves ubiquitin-like molecules from their substrates and is the only known protease specifically deconjugating IFN-stimulated gene 15 (ISG15) [18, 19]. Reports have shown that USP18 is involved in chronic myeloid leukaemia and melanoma by regulating IFN-modulating signaling, indicating its role in cancer-associated immune responses [20, 21]. In addition, dysregulation of USP18 expression leads to IFN-stimulated gene expression in Burkitt lymphoma [22]. In our study, USP18 was also correlated with the type I IFN response, which was consistent with previous studies. Therefore, USP18 might regulate type I IFN-associated immune responses in the development of EN DLBCL.

In addition, we identified that the MAPK pathway was the pathway downstream of USP18 in EN DLBCL. The MAPK pathway participates in various cellular processes, such as cell proliferation, differentiation and apoptosis. It is aberrantly activated in numerous cancers and associated with tumor progression, metastasis and therapy resistance [23, 24]. Knockout of another member of the USP family, USP12, leads to impaired MAPK activity in cells, suggesting that the USP family might regulate the MAPK signaling pathway. Multidimensional validation in our study also showed that a key marker in the MAPK pathway, TLR7, is closely associated with both USP18 expression and prognosis, further indicating the possibility of USP18 regulating the MAPK pathway in EN DLBCL.

Furthermore, we found that USP18 was mostly associated with the immune gene set of aDCs. As antigen-presenting cells, DCs are activated by cytokines to unleash the immune responses of T cells, B cells and NK cells, playing important roles in lymphoma [25]. DCs are reduced in NHL, accompanied by defective DC migration and antigen presentation activity [26, 27]. In pathological tissues of cutaneous T cell lymphoma, a reduced number of DCs was correlated with poor survival [25]. In our study, USP18 was positively correlated with aDC number, indicating less aDC infiltration in the development of EN DLBCL. IHC staining also further confirmed a decreased number of aDCs in EN DLBCL tissues. Moreover, we identified that the immune gene sets of the type I IFN response and Tregs were correlated with USP18 in EN DLBCL. Interestingly, the most important function of DCs is to produce type I IFN [28]. DCs were also reported to promote the expansion and suppressive function of Tregs [29]. Therefore, our study indicated the involvement of USP18 in DC-modulating immune responses in EN DLBCL.

Interestingly, tumor cell-derived proteins could affect the differentiation and function of DCs via the p38 MAPK pathway [30]; thus, we speculated that USP18 might affect DC-modulating immune responses through the MAPK pathway in the development of EN DLBCL. The PPI network generated in String also indicated their interaction. However, this speculation needs further biological experiments for validation.

Of course, *in silico* studies have some limitations. The expression profiles and clinical information used here were from public databases that contain small numbers of samples, and the results were not experimentally confirmed. However, we performed multidimensional validation in several online databases, which lends strong support to the correlations between key biomarkers identified in our analysis. Additionally, we confirmed low expression of USP18 protein and fewer aDCs in the tumor tissues of EN DLBCL patients by IHC staining. Overall, we deduced that USP18 was the key immune gene regulated by LHX2 and affected aDCs and the MAPK pathway, contributing to the development of EN DLBCL. Further experiments will be carried out to confirm our findings.

## CONCLUSIONS

Our results are the first to indicate the potential role of USP18 in EN DLBCL, acting via the MAPK pathway and aDCs. Our findings may provide more clinical information and promising molecular targets for pharmacotherapeutic interventions for EN DLBCL.

## MATERIALS AND METHODS

### Data preparation and analysis of DEGs

Gene expression profiles and clinical characteristics of primary DLBCL samples were downloaded from TCGA (https://portal.gdc.cancer.gov/). HTseq-count and fragments per kilobase of exon per million reads mapped (FPKM) profiles of DLBCL samples, including 25 LN DLBCL and 21 EN DLBCL samples, were assembled. Immune-related genes were collected from the ImmPort database (https://www.immport.org/) [31]. Cancer-related TFs and ChIP-Seq data were retrieved from the Cistrome Cancer database (http://cistrome.org/) [32]. To identify DEGs between LN and EN DLBCL, the edgeR method was applied [33]; P < 0.05 and log (fold change) > 1 or < -1 were set as the cut-offs. Volcano plots and heatmaps were generated to show DEGs. Finally, GO and KEGG enrichment analyses of DEGs were performed to reveal the potential mechanism of EN DLBCL.

### Identification of prognostic immune genes and construction of the predictive model

Volcano plots and heatmaps were created to illustrate the expression of DEIGs, which were extracted from the previous DEG and immune-related gene lists. Then, univariate Cox regression analysis was applied to identify the prognostic immune genes based on DEIGs and clinical information, with cut-offs of P < 0.05 and log (fold change) > 1 or < -1.

To assess the significance of each prognostic immune gene with a β value, which was the regression coefficient of integrated genes in the model, multivariate Cox regression analysis was carried out. The significant factors in the univariate Cox regression analysis were sent to the multivariate Cox regression analysis. The following formula was used to calculate the risk score:

$$\begin{array}{l} \text{Risk Score }=\text{ β}1\;\times \text{DEIG}1+\text{β}2\;\times \text{DEIG}2+\;\\ \text{β}3\;\times \text{DEIG}3\ldots \ldots +\text{βn}\times \text{DEIGn} \end{array}$$

In the formula, “n” is the number of prognostic immune genes in the model. “β” is the regression coefficient of each integrated gene. “DEIG_n_” is the expression level of each integrated gene. Based on the model, patients were reordered and divided into high- and low-risk groups with the median risk score. To avoid model overfitting, Lasso regression and Schoenfeld residuals tests were performed. The AUC was applied to evaluate the accuracy of the model. Kaplan-Meier survival analysis was performed to compare patient survival between the two risk groups. Next, risk curve, survival state-related scatterplot and heatmap of prognostic immune genes were plotted based on the risk score.

### Identification of key immune genes

Volcano plots and heatmaps were created to show the expression of DETFs, which were obtained by intersecting DEGs and cancer-related TFs. Then, to reveal the regulations and associations between DETFs and prognostic immune genes, Pearson correlation analysis was conducted, and only regulatory pairs with a correlation coefficient > 0.300 and P < 0.001 were selected for the next analysis. The intersection of prognostic immune genes in the above regulatory pairs and differentially expressed between EN and LN DLBCL by the Wilcoxon test was performed, as shown in the Venn plot. The immune gene in the regulatory pair with the highest coefficient and differentially expressed between EN and LN DLBCL was recognized as the key immune gene.

### Validation of the regulatory mechanism between the key TF and immune gene

The regulatory mechanism between the key TF and immune gene was verified by ChIP-Seq. Two algorithms (JASPAR [34] and ENCODE transcription factor targets) were utilized to illustrate the transcriptional regulation patterns between LHX2 and USP18 to further confirm our hypothesis. LHX2 ChIP-Seq data from an *in vitro* cell line in the Cistrome database were downloaded to validate the transcriptional regulation patterns of USP18.

### Identification of potential downstream KEGG pathways and immune gene sets

To determine the pathways downstream of key immune genes, GSVA was performed to identify differential KEGG pathways between EN and LN DLBCL. GSVA was implemented using the “gsva” package of R and under default settings except for “RNAseq = TRUE”. The GSVA algorithm accepted input from a gene expression matrix (log2-normalized RNA-seq count data) and a specific set of genes. The final output was a data matrix corresponding to each sample with each gene set. Pathways with P < 0.05 were selected and displayed. Pearson correlation analysis was used to uncover the relationship between the key immune genes and ENI-related signaling pathways, as shown by a coexpression heatmap. GSEA was also used to identify ENI-related signaling pathways [35]. Pathways with P < 0.05 was selected. The overlapping KEGG pathways from both GSEA and GSVA, illustrated by a Venn plot, were recognized as potential downstream pathways. The correlation between key immune genes and potential downstream pathways was fitted by linear regression.

ssGSEA was applied to identify immune responses in DLBCL from 29 immune gene sets that were overexpressed in the tumor microenvironment [11, 36]. Pearson correlation analysis was performed to illustrate the relationship between key immune gene and immune gene sets, as shown by the coexpression heatmap. Immune gene sets with P < 0.05 were selected and displayed. The top three correlations between key immune gene and immune gene sets were fitted by linear regression.

### Online database validation and construction of regulation network including key TF, immune gene, KEGG pathways, and immune gene sets

For further annotation of identified TF, biomarker, immune gene sets, and signaling pathways, several online databases were used to detect gene and protein expression level. UALCAN [37], UCSC xena [38], Linkedomics [39], Gene Expression Profiling Interactive Analysis (GEPIA) [40], cBioportal [41] and Oncomine [42] were applied to validate the association between gene expression and clinical significance in tissue level in DLBCL. Furthermore, we used Cancer Cell Line Encyclopedia (CCLE) [43] to verify the gene expression in cellular level in DLBCL. Then the human protein altas [44] were applied to show the protein expression level in normal tissue. Finally, String [45] displayed the interaction network among LHX2, USP18 and the downstream pathway.

To show our results more clearly, a network based on the interaction among key TF, immune gene, KEGG pathways and immune gene sets was built by Cytoscape 3.7.1 [46]. Finally, EN DLBCL- related hypothesis built on the bioinformatics was displayed by signaling diagram.

### Immunohistochemistry and immunofluorescence double staining

IHC and immunofluorescence staining were conducted according to standard methods on EN and LN DLBCL biopsies. Briefly, 5-μm formalin-fixed and paraffin-embedded (FFPE) sections were deparaffinized and hydrated. The sections were incubated overnight at 4° C in a humidified slide chamber with primary antibodies against USP18 (1:200, ab115618, Abcam) and CD83 (1:50, ab205343, Abcam). Finally, to assess the percentage of positive tumor cells, all the IHC slides were viewed and given a histochemistry score (H-score).

H −score = ∑pi(i + 1)

i represents the intensity score, and pi is the percentage of cells with that intensity. All immunofluorescence slides were observed with a confocal laser scanning microscope.

### Statistical analysis

For descriptive statistics, the continuous variables in normal distribution were expressed as mean ± standard deviation (SD) while the median (range) was used in abnormal distribution. Classified variables were expressed by counts and percentages. Only two-tailed P < 0.05 was considered statistically significant. All statistical analysis was performed using R version 3.5.1 (Institute for Statistics and Mathematics, Vienna, Austria; https://www.r-project.org).

### Ethical review committee statement

This study was approved by the Ethics Committee of Shanghai Ninth People’s Hospital, Shanghai Jiao Tong University School of Medicine.

## Supplementary Material

Supplementary Figures

Supplementary Table 1
