# Supplementary material for: Downregulation of USP18 reduces tumor-infiltrating activated dendritic cells in extranodal diffuse large B cell lymphoma patients
Source: Aging (Albany NY). 2021 May 17;13(10):14131–58. doi: 10.18632/aging.203030 (PMC8202869; doi:10.18632/aging.203030)
Supplement: Supplementary Figures [file aging-13-203030-s001.pdf]

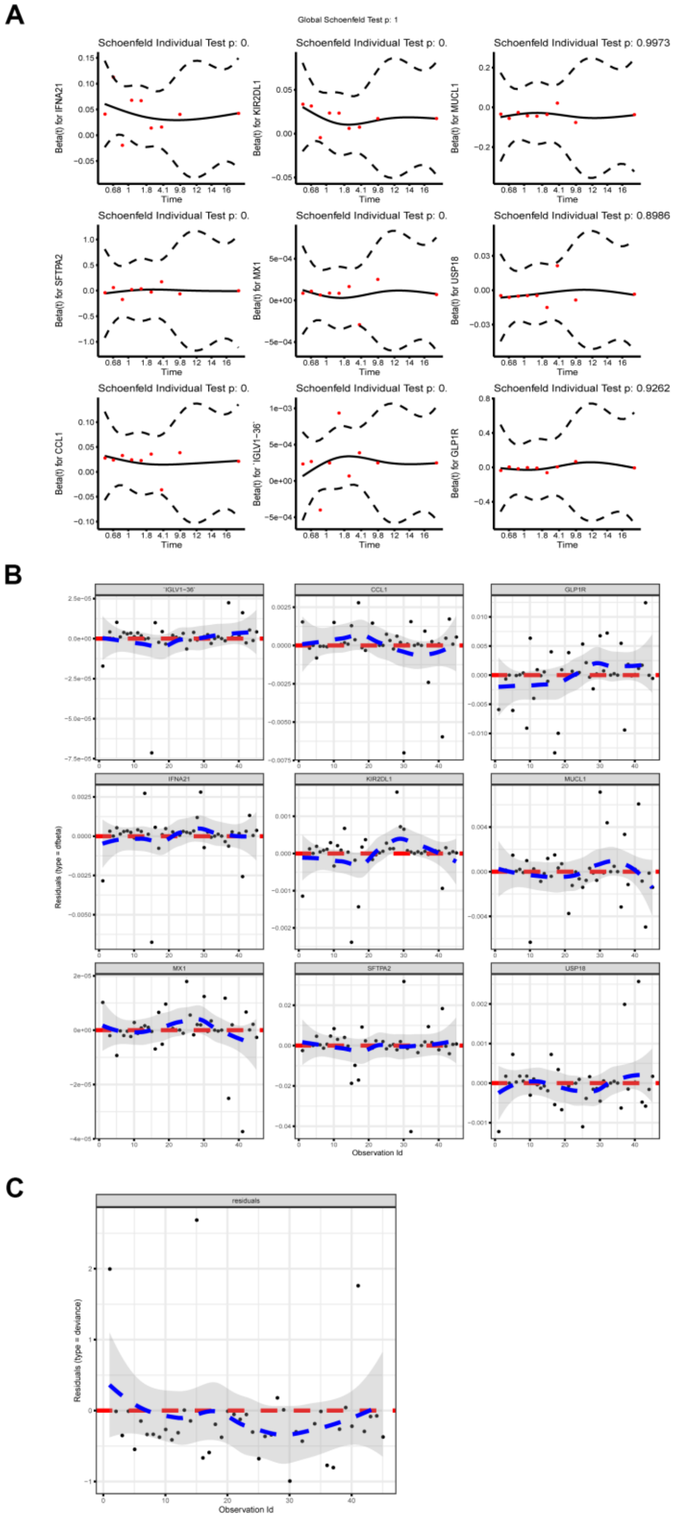

Supplementary Figure 1. The result of Schoenfeld residuals test. (**A**) The Schoenfeld residuals test of each key immune gene. (**B**) The residuals plot of each key immune gene. (**C**) The residuals plot of multivariable Cox regression analysis.

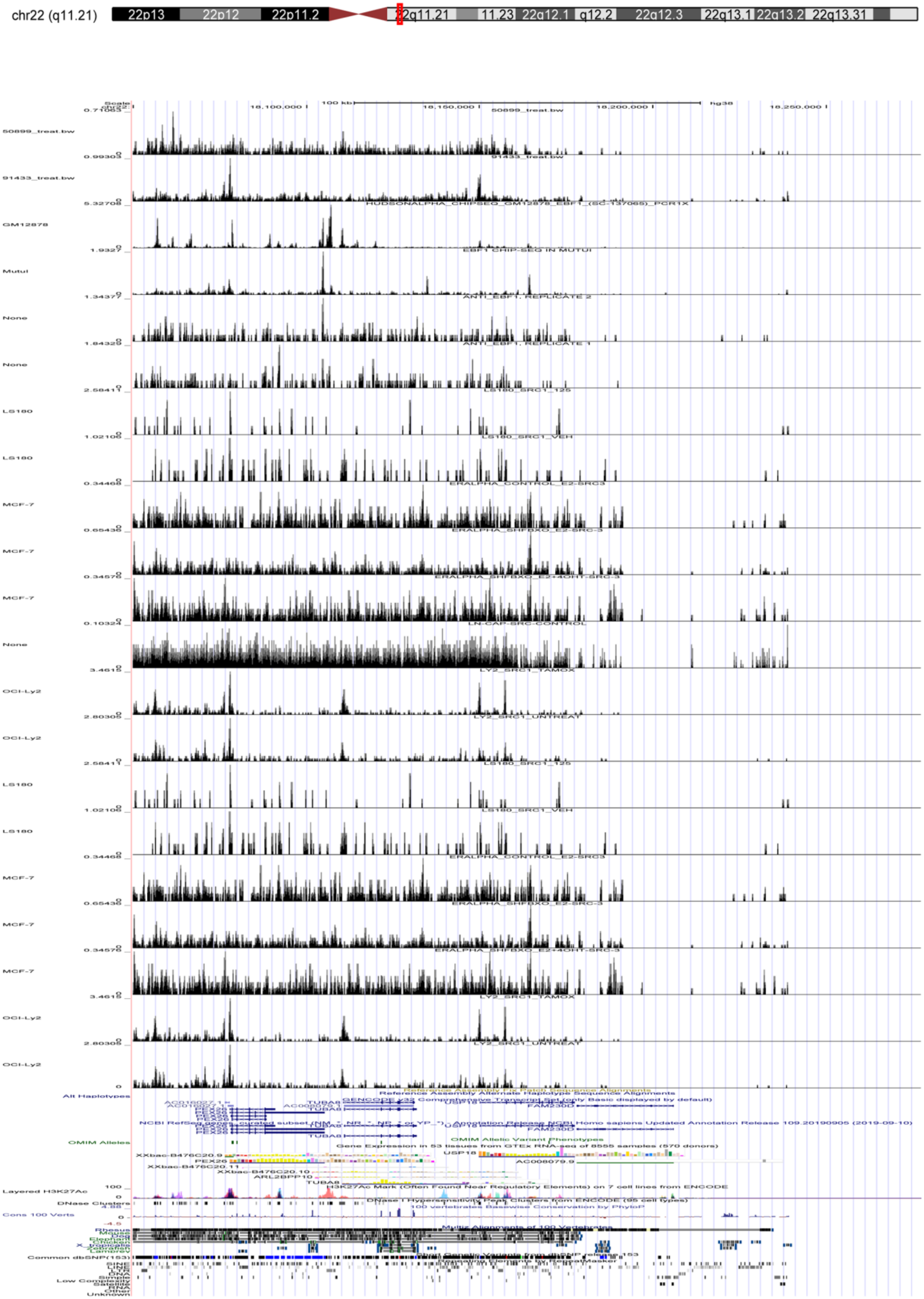

Supplementary Figure 2. The validation of regulatory mechanism between LHX2 and USP18 based on ChIP-Seq data from Cistrome database.

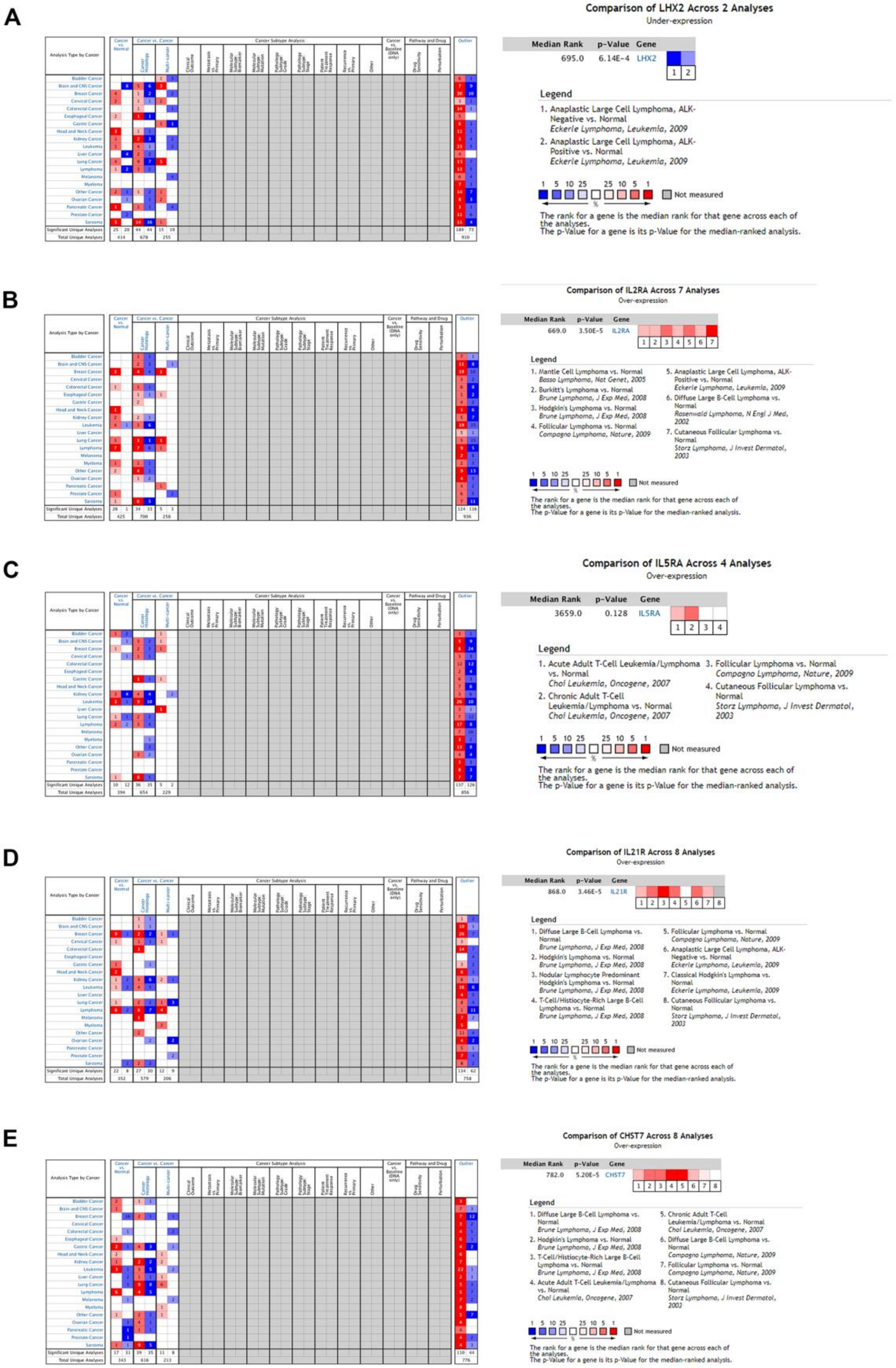

Supplementary Figure 3. Validation of LHX2, IL2RA, IL5RA, IL21R and CHST7 (**A**–**E**) on the transcriptional level in multiple cancer types and multiple studies from the Oncomine database.

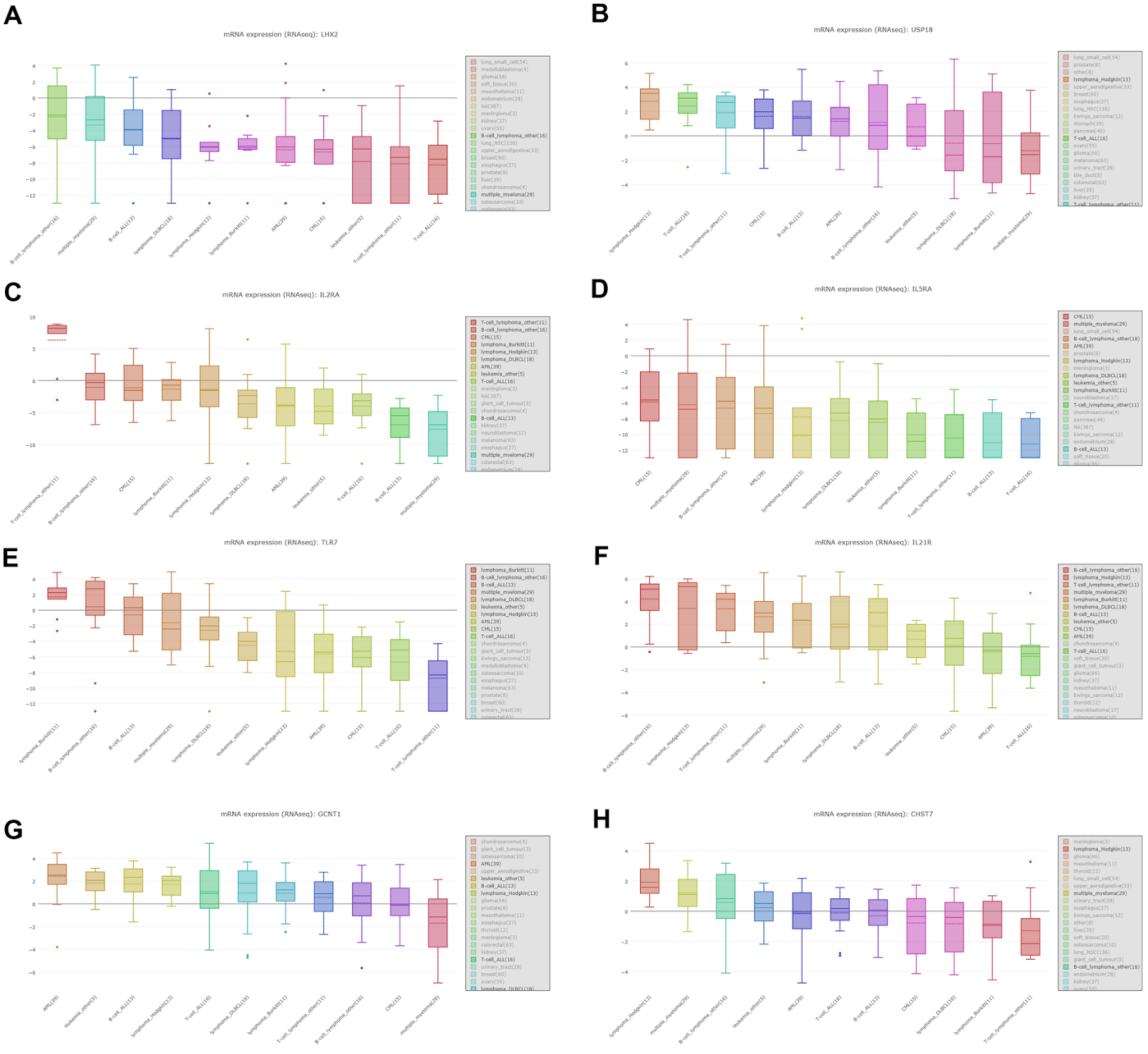

Supplementary Figure 4. The expression levels of LHX2, USP18, IL2RA, IL5RA, TLR7, IL21R, GCNT1 and CHST7 (**A**–**H**) in various hematologic malignancy cell lines in Cancer Cell Line Encyclopedia (CCLE).

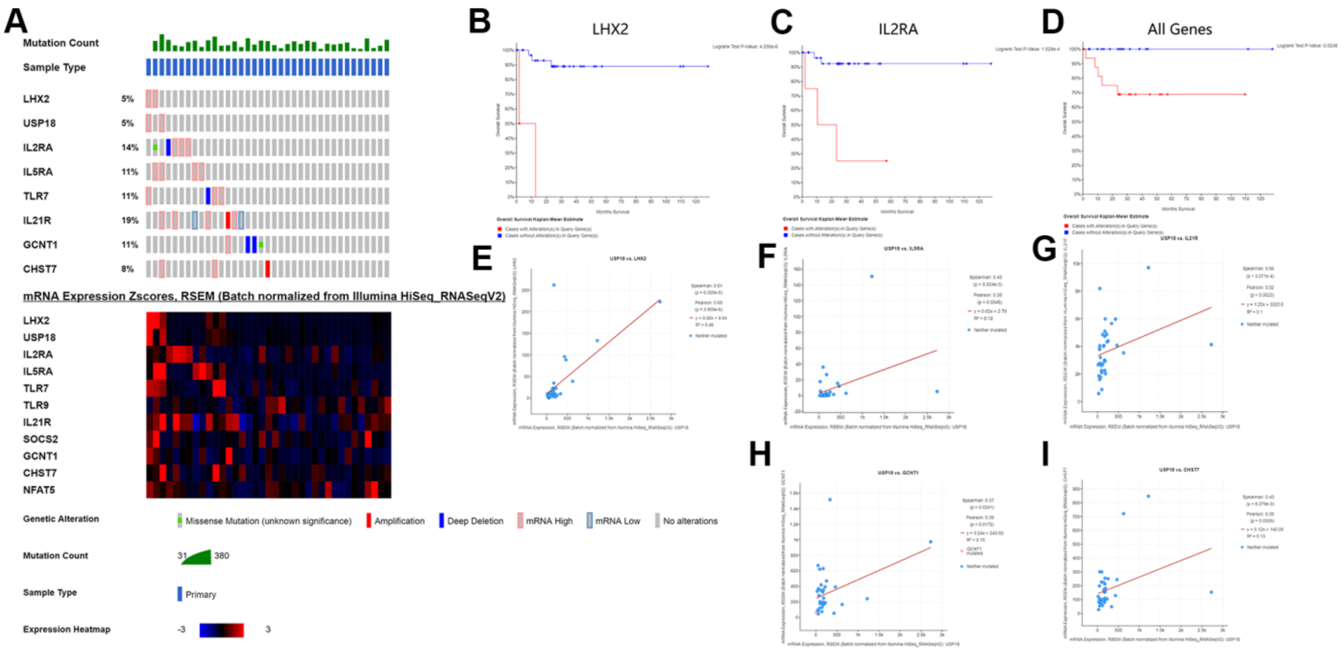

Supplementary Figure 5. Integrative analysis of genomics and clinical profiles with the cBioPortal database. (**A**) Alteration frequency of LHX2, USP18, ILL2RA, IL5RA, TLR7, IL21R, GCNT1 and CHST7; (**B**–**D**) Kaplan-Meier survival curves of LHX2, IL2RA and all genes; (**E**–**I**) The co-expression between USP18 and LHX2, IL5RA, IL21R, GCNT1 and CHST7.

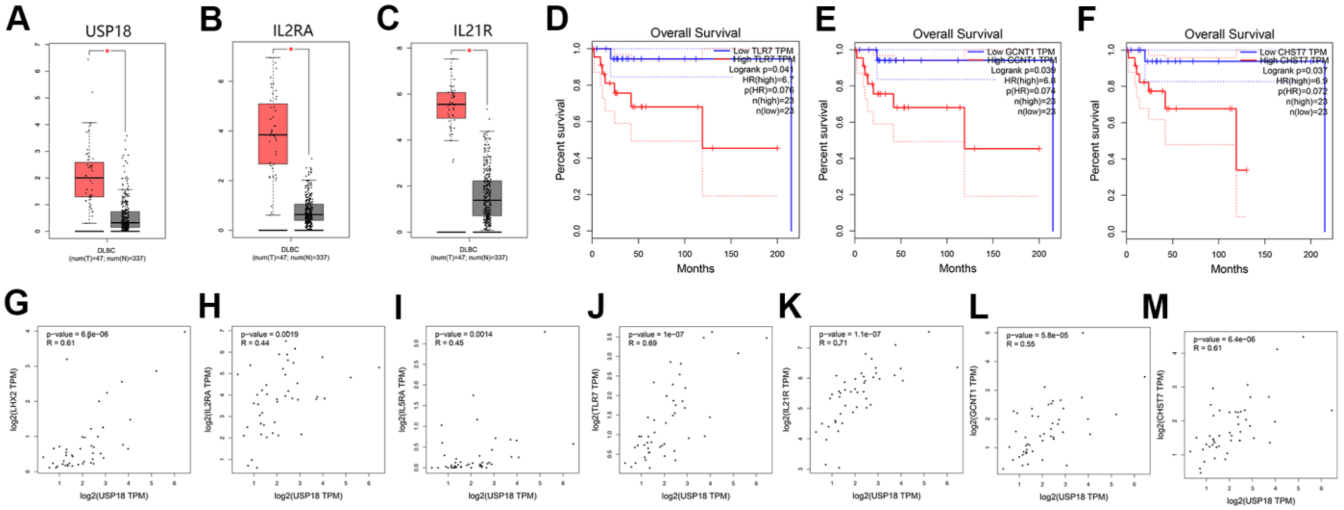

Supplementary Figure 6. Integrative analysis of transcriptional and clinical profiles using the GEPIA database. (**A**–**C**) The expression levels of USP18,IL2RA and IL21R between normal samples and DLBCL samples; (**D**–**F**) Kaplan-Meier survival curves of TLR7, GCNT1 and CHST7; (**G**–**M**) The co-expression between USP18 and LHX2, IL2RA, IL5RA, TLR7, IL21R, GCNT1 and CHST7.

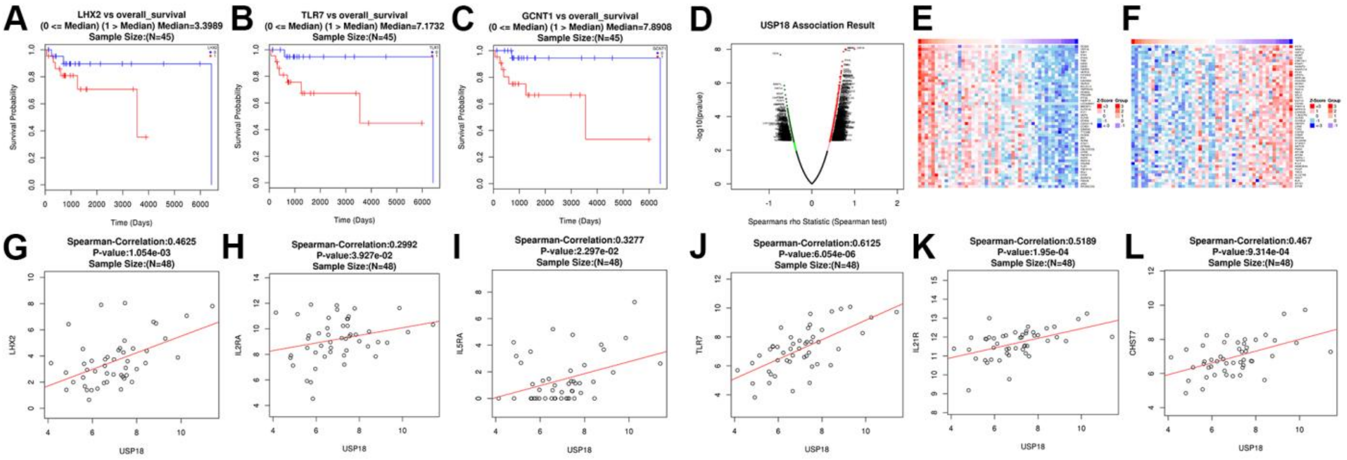

Supplementary Figure 7. Evaluation prognostic value and relationship with USP18 of all the markers identified in the LinkedOmics database. (**A**–**C**) Kaplan-Meier survival curves of LHX2, TLR7 and GCNT1; (**D**–**F**) The correlation relationship between USP18 and proteins in RPPA; (**G**–**L**) The co-expression between USP18 and LHX2, IL2RA, IL5RA, TLR7, IL21R and CHST7.

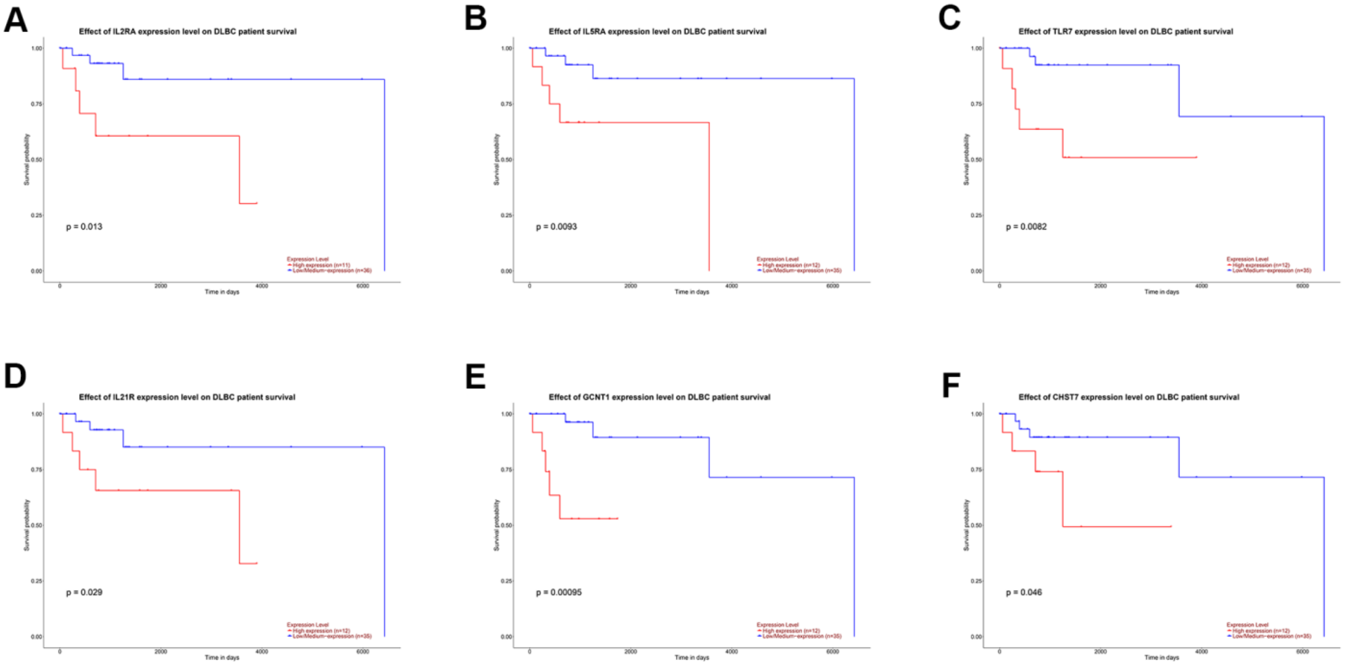

Supplementary Figure 8. Kaplan–Meier survival curves of IL2RA, IL5RA,TLR7, IL21R, GCNT1 and CHST7 (**A**–**F**) using the UALCAN database.

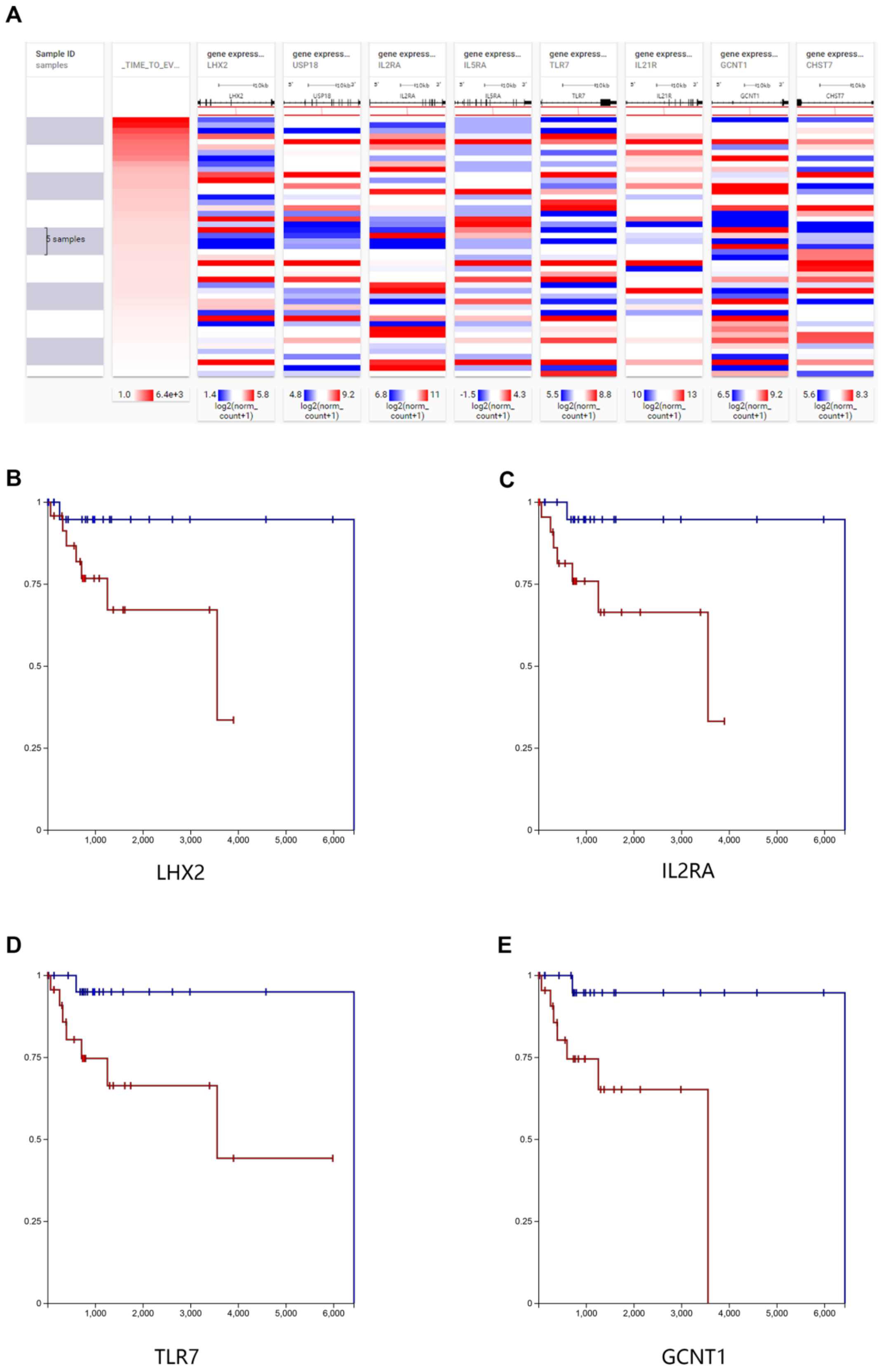

Supplementary Figure 9. Integrative analysis of genomics and clinical profiles with the UCSC xena database. (**A**) The expression level of LHX2, USP18, IL2RA, IL5RA, TLR7, IL21R, GCNT1 and CHST7 in DLBCL; (**B**–**E**) Kaplan-Meier survival curves of LHX2, IL2RA, TLR7 and GCNT1.

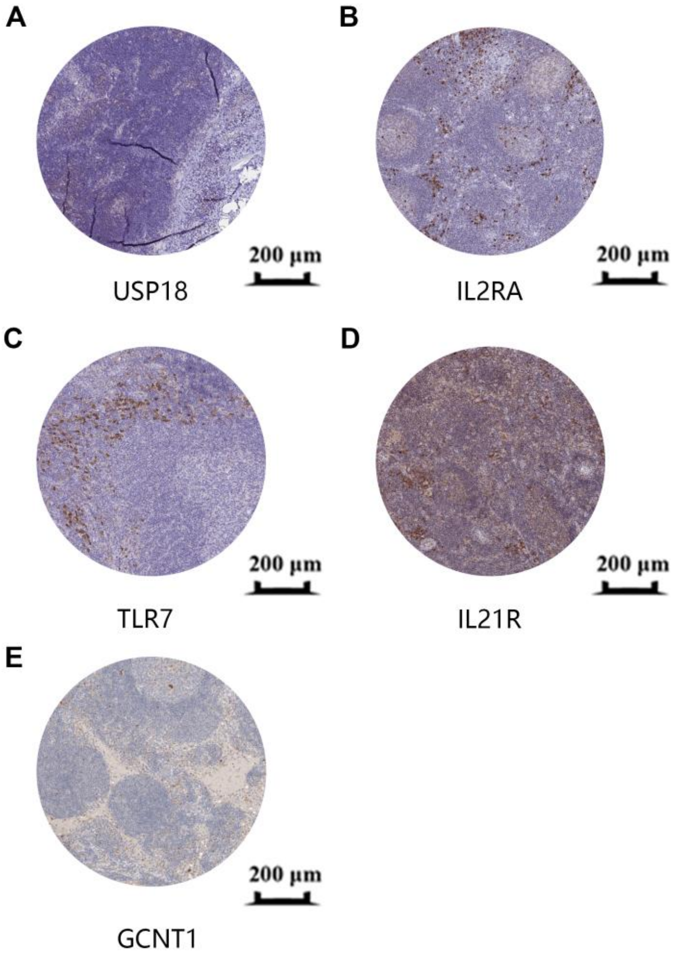

Supplementary Figure 10. Validation of USP18, IL2RA, TLR7, IL21R and GCNT1 (**A**–**E**) in the normal lymph node tissue on a translation level in the Human Protein Atlas database.

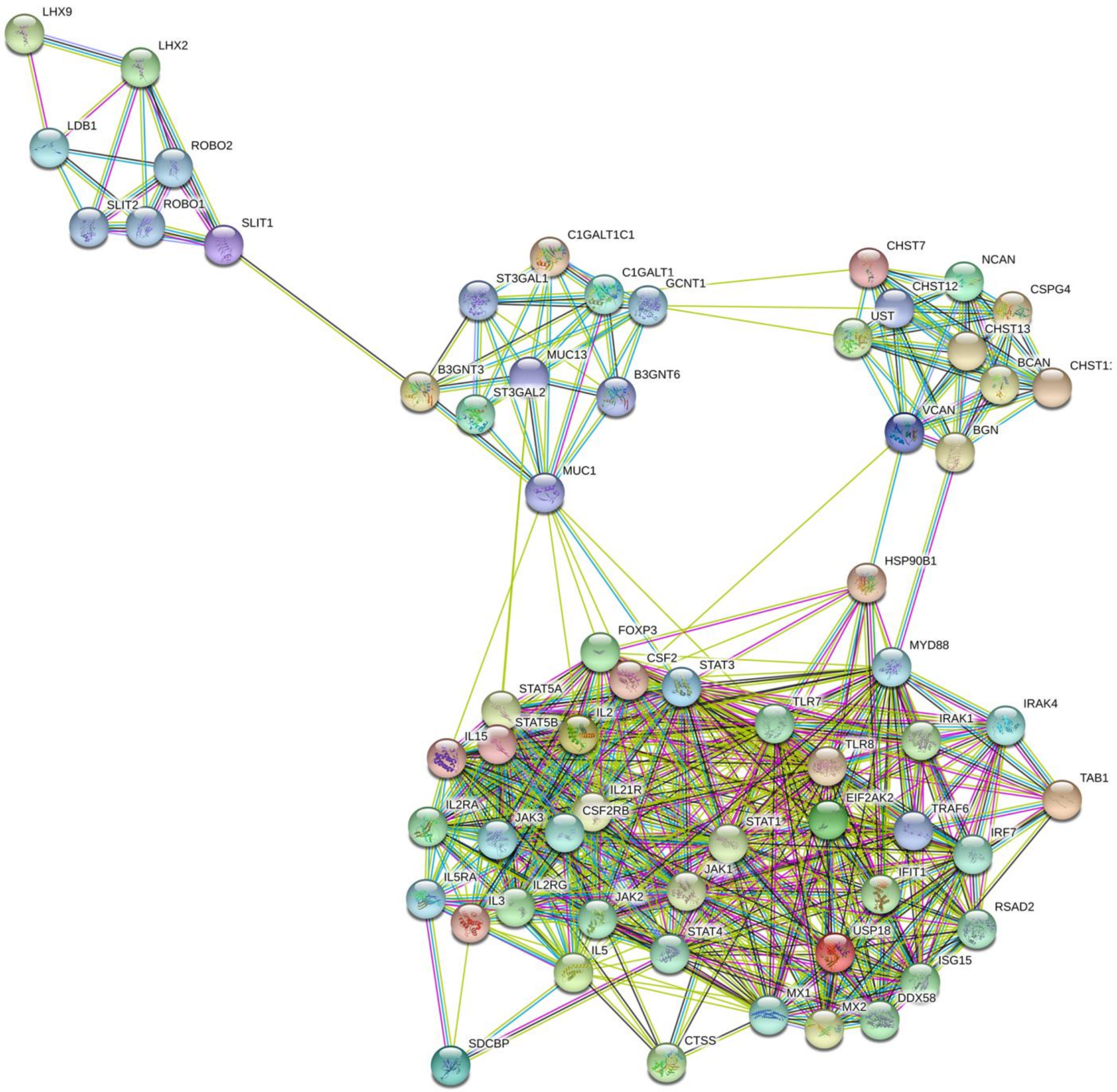

Supplementary Figure 11. The protein-protein interaction (PPI) network of all the markers in string.

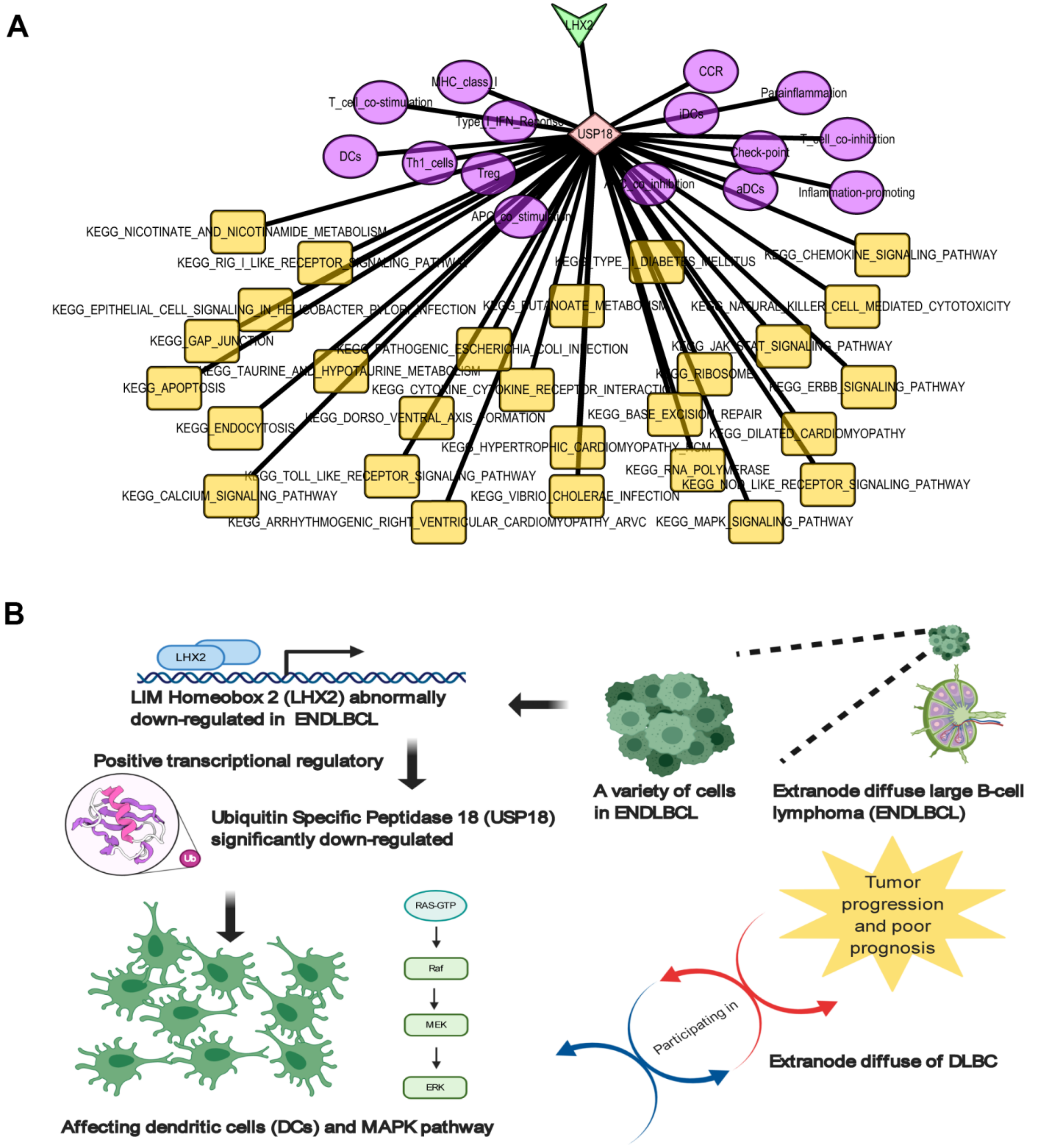

Supplementary Figure 12. The network of this scientific hypothesis including key TF, immune gene, downstream KEGG pathways and immune gene sets. (**A**) The protein-protein interaction network among key TF, immune gene, KEGG pathways and immune gene sets by Cytoscape; (**B**) The schematic diagram of this scientific hypothesis.
