# Supplementary material for: Downregulation of USP18 reduces tumor-infiltrating activated dendritic cells in extranodal diffuse large B cell lymphoma patients
Source: Aging (Albany NY). 2021 May 17;13(10):14131–58. doi: 10.18632/aging.203030 (PMC8202869; doi:10.18632/aging.203030)
Supplement: Supplementary Table 1 [file aging-13-203030-s002.docx]

**Supplementary Table 1 External validation of key biomarkers via multiple online databases.**

|  | LHX2 (oncogene) | USP18(oncogene) | IL2RA (oncogene) | IL5RA (oncogene) | TLR7 (oncogene) | IL21R (oncogene) | GCNT1 (oncogene) | CHST7 (oncogene) |
| --- | --- | --- | --- | --- | --- | --- | --- | --- |
| UALCAN | K-M: P = 0.94 | K-M: P = 0.17 | K-M: P = 0.013 | K-M: P = 0.009 | K-M: P = 0.008 | K-M: P = 0.029 | K-M: P < 0.001 | K-M: P = 0.046 |
| UCSC Xena | K-M: P = 0.036 | K-M: P = 0.240 | K-M: P = 0.021 | K-M: P = 0.146 | K-M: P = 0.026 | K-M: P = 0.146 | K-M: P = 0.008 | K-M: P = 0.081 |
| LinkedOmics | K-M: P = 0.048  Correlation: R = 0.463  P = 0.001 | K-M: P = 0.106 | K-M: P = 0.077  Correlation: R = 0.300  P = 0.039 | K-M: P = 0.137  Correlation: R = 0.328  P = 0.023 | K-M: P = 0.031  Correlation: R = 0.613  P < 0.001 | K-M: P = 0.229  Correlation: R = 0.519  P < 0.001 | K-M: P = 0.016  Correlation: R = 0.282  P = 0.052 | K-M: P = 0.278  Correlation: R = 0.467  P < 0.001 |
| GEPIA | K-M: P = 0.160  Expression: P > 0.05  Correlation: R = 0.61  P < 0.001 | K-M: P = 0.260  Expression: P < 0.05 | K-M: P = 0.130  Expression: P < 0.05  Correlation: R = 0.44  P = 0.002 | K-M: P = 0.280  Expression: P > 0.05  Correlation: R = 0.45  P = 0.001 | K-M: P = 0.041  Expression: P > 0.05  Correlation: R = 0.69  P < 0.001 | K-M: P = 0.11  Expression: P < 0.05  Correlation: R = 0.71  P < 0.001 | K-M: P = 0.039  Expression: P > 0.05  Correlation: R = 0.55  P < 0.001 | K-M: P = 0.037  Expression: P > 0.05  Correlation: R = 0.61  P < 0.001 |
| cBioportal | Correlation: R = 0.609  P < 0.001  K-M: P < 0.001 | K-M: P = 0.210 | Correlation: R = 0.085  P = 0.615  K-M: P < 0.001 | Correlation: R = 0.446  P = 0.006  K-M: P = 0.546 | Correlation: R =0.517  P = 0.001  K-M: P = 0.674 | Correlation: R = 0.559  P < 0.001  K-M: P = 0.786 | Correlation: R = 0.370  P = 0.024  K-M: P = 0.533 | Correlation: R = 0.428  P = 0.008  K-M: P = 0.354 |
| Human protein atlas | NA | Normal: Not detected | Normal: Medium | NA | Normal: Not detected | Normal: Medium | Normal: Low | NA |

Abbreviations: LHX2, LIM Homeobox 2; USP18, Ubiquitin Specific Peptidase 18; IL2RA, Interleukin 2 Receptor Subunit Alpha; IL5RA, Interleukin 5 Receptor Subunit Alpha; TLR7, Toll Like Receptor 7; IL21R, Interleukin 21 Receptor; GCNT1, Glucosaminyl (N-Acetyl) Transferase 1; CHST7, Carbohydrate Sulfotransferase 1.
